# Targeting risk factors and strategies for prevention of peripheral neuropathy: focus on B-vitamin deficiencies and metabolic diseases

**DOI:** 10.1186/s42466-026-00466-8

**Published:** 2026-04-01

**Authors:** Petra Baum, Joanna Kosacka, Thomas Ebert

**Affiliations:** 1 Department of Neurology, University of Leipzig, Liebigstr. 20, Leipzig, 04103 Germany; 2 Department of General, Visceral and Vascular Surgery, Jena University Hospital, Jena, 07747 Germany; 3 Medical Department III - Endocrinology, Nephrology, Rheumatology, University of Leipzig, Liebigstr. 20, Leipzig, 04103 Germany

**Keywords:** Diabetes mellitus, Idiopathic polyneuropathy, Inflammation, Metabolic syndrome, Peripheral neuropathy, Prevention strategies, Vitamin b deficiency

## Abstract

**Background:**

Polyneuropathy (PN) is a common neurological disorder characterized by sensory, motor, and autonomic dysfunction of peripheral nerves. Its incidence is increasing, especially among the elderly population, making PN a significant and growing public health concern worldwide.

**Main body:**

This review highlights the key risk factors contributing to the development of PN, with a particular focus on nutritional and metabolic etiologies. These include B-vitamin deficiencies, prediabetes, diabetes mellitus, obesity, and treatment-induced neuropathies. The complex interplay of these factors often exacerbates the progression of PN and complicates management. Current preventive approaches and challenges in early detection are also discussed to provide a comprehensive overview of strategies aimed at reducing the disease burden.

**Conclusion:**

The rising prevalence and disabling nature of PN necessitate urgent public awareness, better screening, and increased healthcare resources. Advancing prevention strategies targeting modifiable risk factors may significantly reduce the impact of PN on patients and healthcare systems alike.

## Background

Polyneuropathy (PN) is a peripheral neuropathy characterized by sensory, motor, and/or autonomic dysfunction of nerve fibers. It typically presents as symmetrical sensory symptoms such as paresthesia, pain and muscle weakness, predominantly affecting the distal regions of the arms and legs.

The prevalence of PN in the general population is estimated to be around 1%, increasing to approximately 30% in the elderly, with a further rise expected in the coming years [1, 2]. This growing burden is of particular concern, as PN is a disabling condition that severely affects quality of life. Affected individuals often experience both sensory and motor impairments, which contribute to an increased risk of falls, functional limitations, amputations, and other medical complications [3, 4]. Despite its impact, PN often remains underrecognized by both patients and physicians [3, 5].

Beyond its impact on individual patients, PN constitutes a substantial public health burden. Population-based studies have shown that individuals with PN frequently face reduced work capacity, loss of independence, chronic pain requiring multiple medications, and even shortened life expectancy [3]. Therefore, greater public attention and healthcare resources are needed to address PN as a serious health issue and to develop effective prevention strategies.

Multiple risk factors have been identified in the development and progression of PN. The most common include diabetes mellitus (30%−50%), vitamin deficiencies (15%), alcohol abuse, medication, or a combination of various risk factors (20%) [3, 5, 6].

Nevertheless, up to 20%–30% of PN cases remain idiopathic, with no identifiable underlying cause [1]. This significant proportion suggests that certain risk factors may still be underrecognized, inadequately screened, or poorly understood. Genetic predispositions and subtle nutritional or metabolic imbalances may play a role in these unexplained cases. Future studies are needed to assess the risks for associated diseases and to identify new, potentially modifiable risk factors involved in the development and progression of PN. In this context, metabolic neuropathies represent a particularly important subgroup. These neuropathies are commonly linked to systemic metabolic dysfunctions, with diabetes mellitus, metabolic syndrome, and micronutrient deficiencies, especially involving B-vitamins, being among the most prominent causes.

This article, therefore, aims to highlight the key risk factors in the development of PN, with a focus on nutritional and metabolic etiologies, and the current state of prevention.

A systematic literature search was performed in PubMed, Embase, Web of Science, and Cochrane CENTRAL up to December 2025. Search terms included: “diabetic peripheral neuropathy”, “vitamin B12 deficiency”, “vitamin B6 deficiency”, “vitamin B1”, “vitamin B2”, “vitamin B9”, “prevalence”, “serum levels”, and “metformin”. Observational studies and interventional trials reporting B‑vitamin status and neuropathic outcomes in patients with and without diabetes were included. Studies not reporting relevant outcomes, animal studies, or single case reports were excluded.

## PN secondary to vitamin B deficiencies

Food deficiency is a global problem influenced by socioeconomic and geographical factors, ranging from food shortages in developing countries to Western dietary patterns characterized by obesity, alcohol misuse, and restrictive eating behaviors such as vegan diets.

Diet-related PN remains underrecognized by general practitioners and neurologists, particularly in individuals who are not overtly malnourished. Thus, individuals with a normal or even elevated body mass index may still be at risk if their diet is limited to only a few essential food groups. In such cases, micronutrient deficiencies may be masked by an otherwise adequate caloric intake, leading to delayed diagnosis and treatment.

In contrast, malnourished individuals frequently present with PN caused by multiple concurrent micronutrient deficiencies, most notably involving the B vitamin complex (e.g., thiamine, pyridoxine, folate, and cobalamin). The clinical spectrum is broad, ranging from solely sensory neuropathy characterized by areflexia, ataxia, and neuropathic pain to motor axonal or mixed sensorimotor axonal PN [7].

Notably, in cases of acute nutritional axonal neuropathy, specific micronutrient deficiencies or identifiable risk factors do not reliably predict the PN subtype. As reported by Hamel and Logigian, the phenotypic variability and lack of correlation with individual deficiencies underscore the complexity of underlying mechanisms and the need for comprehensive nutritional and neurological assessment [7].

### Vitamin B12 (cobalamin)

Vitamin B12 deficiency is an important risk factor for PN, identified by low serum B12 levels accompanied by increased B12-related metabolites, methylmalonic acid (MMA), and/or homocysteine (Hcy) [8]. A so-called “functional” vitamin B12 deficiency, with elevated MMA and/or Hcy levels despite normal B12 serum levels, can already induce a B12-related PN [9].

Vitamin B12 plays a role in cellular DNA/RNA synthesis across all tissues and maintenance of the myelin sheath. The pathogenesis of B12-associated neuropathy is thought to involve the accumulation of neurotoxic metabolites, such as MMA and Hcy, which disrupt neuronal function and myelin synthesis [10].

Macrocytosis in routine blood tests, i.e. complete blood count, is a non-specific pathological indicator of B12 deficiency. More specific biomarkers of vitamin B12 status are serum B12 (serum cobalamin) and holotranscobalamin (holoTC or active B12), which are recommended for first-line testing. If first-line results are indeterminate, second-line testing with MMA and Hcy should be considered [11].

The diagnosis of vitamin B12 deficiency is partly laboratory- and manufacturer-dependent.

At present, there is no “gold standard” biomarker for determining vitamin B12 status. MMA is very commonly used as an index test for vitamin B12 deficiency, with some laboratories reporting a serum MMA concentration > 280 nmol/L as an indicator of suboptimal vitamin B12 status in patients < 65 years of age with normal renal function [11]. Interpretation is difficult in older patients with impaired renal function.

A large decrease in MMA concentration after treatment with B12 is considered confirmation of a pre-existing vitamin B12 deficient state [11].

To date, no systematic studies have investigated the prevalence of vitamin B12 deficiency associated with PN.

.

A pilot study with 38 participants used a cross-sectional design to investigate the relationship between peripheral neuropathy and vitamin B12 status [12]. Participants with adequate vitamin B12 status scored 10%–20% higher in tests of hand dexterity, vibration sensitivity, and balance than participants with borderline to deficient vitamin B12 status (26% of participants with serum vitamin B12 < 221 pmol/L) and provided initial evidence of peripheral neuropathy in individuals with marginal-to-deficient vitamin B12 status [12]. These findings align with population-based data showing a dose-response relationship, in which lower vitamin B12 levels (< 260 pg/mL) are associated with impaired sensory and motor nerve function, independent of diabetes [13].

A large study on the prevalence of PN in the general population of middle-aged and elderly people showed that vitamin B12 deficiency was a possible cause in 14% of all cases with definite polyneuropathy and in 17% of cases with newly diagnosed PN [5]. Further supporting these findings, Warendorf et al. [14] demonstrated that metabolic vitamin B12 deficiency can be clinically relevant even when serum B12 levels are normal. Following vitamin B12 supplementation, 19% of patients showed clinical improvement, while 24% showed milder neuropathic symptoms; most responders had absolute or metabolic vitamin B12 deficiency.

A high prevalence of B12 deficiency is found in various patient groups, e.g., patients after bariatric or metabolic surgery (30%), patients with malabsorptive diseases (6%−38%), or those taking certain medications, such as antacids and metformin (14%−23%) [15–18].

Additionally, the prevalence of vitamin B12 deficiency among adult vegans can be as high as 52% [19]. Therefore, it is important to focus on the B12 status of patients with the mentioned pre-existing diseases and medications.

A significant issue associated with long-term metformin treatment in patients with diabetes mellitus is its impact on vitamin B12 absorption. Metformin has been shown to reduce the active, intrinsic factor-dependent absorption of vitamin B12 in the intestine. A meta-analysis indicated that patients with T2D on metformin treatment have twice the risk of developing vitamin B12 deficiency compared to those not treated with the medication [20].

Moreover, there is a general lack of awareness among neurologists regarding the decrease in vitamin B12 levels in patients with Parkinson’s disease, which is attributed to the increased methylation demands associated with L-Dopa therapy. This deficiency can contribute to the development of PN in affected individuals [21, 22].

A high prevalence of PN up to 30% was found in non-diabetic older adults in two US cohorts, in which the underlying etiology remained unclear. However, the vitamin B status was not recorded [2].

On the other hand, age is an important risk factor for vitamin B12 deficiency. The 2017 KORA study found that approximately 25% of individuals over 65 and more than a one third of those aged 85–93 had low vitamin B12 levels [23].

Possible causes of B12 deficiency in elderly individuals include chronic gastritis or inadequate food intake, especially a lower consumption of animal-based foods such as meat and fish.

Among vegans, B12 deficiency prevalence may reach 52% [19]. A systematic review and meta-analysis by Niklewicz and coauthors revealed significantly lower serum B12, elevated Hcy and MMA, as well as reduced holoTC, levels in vegans compared to omnivores, indicating both low and functional B12 deficiency. Subgroup analyses showed that the use of vitamin B12 supplements among vegans leads to significant improvements in all biomarker concentrations compared to those who do not take supplements [24]. Notably, dairy and egg consumption in lacto-ovo vegetarians does not confer sufficient protection against B12 deficiency [25]. Thus, neither dairy products nor eggs provide a significant benefit on vitamin B12 status. In accordance with these studies, Arnold and Johnston demonstrated a correlation between low B12 status and impaired hand dexterity, vibration sensitivity, and balance in young vegetarians, suggesting early neuropathic changes [12].

While plant-based diets might offer some metabolic benefits, their low B12 content necessitates supplementation to prevent neurological complications. Therefore, vitamin B12 supplementation is recommended for healthy vegans and vegetarians, and supplemented vegans and vegetarians show comparable B12 status to omnivores [24, 26].

### Vitamin B9 (Folate)

Folate (vitamin B9) deficiency is a further contributor to peripheral neuropathy, with a pathophysiological profile comparable to that of vitamin B12 deficiency. A UK-based population study by Taverner et al. found that folate deficiency and insufficiency were significantly associated with an increased risk of peripheral neuropathy, particularly in individuals under 40 years of age [27].

Like vitamin B12, folate is an essential nutrient that cannot be synthesized endogenously and must be obtained through dietary intake [10]. Rich dietary sources of folate include green leafy vegetables, legumes, broccoli, and cauliflower. However, folate content is highly sensitive to industrial processing, prolonged cooking, and storage conditions, which can substantially reduce its bioavailability. The most common cause of B9 deficiency is lack of dietary intake. Additional causes of folate deficiency include malabsorptive disorders (e.g., celiac disease), chronic alcohol use, certain medications (e.g., sodium valproate and folate antagonists, such as methotrexate), as well as increased physiological demand, as seen in pregnancy and hematological disorders [10].

Interestingly, vegetarians and especially vegans typically exhibit elevated folate levels due to their plant-rich diets. A study by Gallego-Narbón et al. confirmed that Spanish vegans had significantly higher erythrocyte folate concentrations than both vegetarians and omnivores [19, 25]. Similarly, Gilsing et al. (2010) reported that vegans had the highest serum folate levels among British males, despite concurrently showing the highest prevalence of vitamin B12 deficiency [19, 25].

Genetic factors also affect folate metabolism and may contribute to folate deficiency. The enzyme 5,10-methylenetetrahydrofolate reductase (MTHFR) catalyzes the conversion of folate to its biologically active form, 5-methyltetrahydrofolate (5-MTHF), which is essential for the re-methylation of homocysteine to methionine. A common polymorphism in the *MTHFR* gene (677 C→T), present in a significant proportion of the European population, is associated with reduced 5-MTHF production and elevated plasma homocysteine levels [28].

Emerging evidence suggests that supplementation with 5-MTHF may raise plasma folate levels more effectively than synthetic folic acid, even in individuals with *MTHFR* polymorphisms, and may serve as a viable alternative to standard folic acid supplementation [29].

### Vitamin B1 (Thiamine)

B1 deficiency can present as isolated PN, as Wernicke’s encephalopathy, or as a combination of both [10].

Patients with isolated B1 deficiency developed an acute, motor predominant PN with minimal sensory involvement. In contrast, patients with alcohol-induced neuropathy more often develop a slowly progressive, painful sensory neuropathy [30]. However, recent findings suggest that specific micronutrient deficiencies or associated risk factors do not reliably predict the neuropathy subtype (sensory, motor, or mixed) in cases of acute nutritional axonal neuropathy [7].

Vitamin B1 must be obtained almost entirely through dietary intake, as it cannot be synthesized by the body. Dietary sources of thiamine are diverse and include both animal (e.g. pork and fish, such as salmon) and plant-based foods (e.g. seeds and nuts, black beans, soybeans). Due to its short biological half-life and limited tissue storage, a regular dietary supply is essential to maintain adequate thiamine levels [32].

Chronic alcoholism is one of the most common causes of thiamine deficiency worldwide. Alcohol interferes both with intestinal absorption and with the phosphorylation of thiamine into its active form, thiamine pyrophosphate [10].

Additional risk factors for B1 deficiency include a monotonous diet with starchy, thiamine-poor staple foods such as polished rice or cassava (manioc), and the habitual consumption of thiamine antagonists found in foods like betel nuts and certain teas [31].

Infants are especially vulnerable to the consequences of thiamine deficiency, particularly in the first months of life. Exclusively breastfed infants of thiamine-deficient mothers are at highest risk [32].

Patients who have undergone bariatric or metabolic surgery, are also at an increased risk of B1 deficiency due to impaired nutrient absorption. Thiamine requirements are also elevated in critically ill patients, those receiving intensive medical care, and individuals receiving intravenous glucose solutions [10, 31].

### Vitamin B6 (pyridoxine)

Vitamin B6 is a cofactor for two enzymes involved in homocysteine metabolism. Deficiency can lead to increased plasma homocysteine levels, which result in PN and optic neuropathy [10].

Nutritional vitamin B6 deficiency is rare and typically occurs only in cases of severe malnutrition, chronic alcohol abuse, or as a result of medications that interfere with B6 metabolism, such as isoniazid, hydralazine, penicillamine, and levodopa/carbidopa [10, 33].

Paradoxically, peripheral neuropathy may also result from high doses of vitamin B6 supplements. This is primarily due to pyridoxine, the inactive form of vitamin B6, which can competitively inhibit the active form, pyridoxal-5′-phosphate, thereby inducing a functional B6 deficiency [10]. Consequently, symptoms of pyridoxine-induced neuropathy often mimic those of true B6 deficiency and manifest predominantly as sensory axonal neuropathy [34, 35]. Thus, chronic supplementation with pyridoxine at doses exceeding 50 mg/day has been linked to toxic neuropathy and should, therefore, be avoided in clinical practice [33].

### Vitamin B2 (riboflavin)

Vitamin B2 is essential for normal cellular metabolism. Its derivatives, flavin mononucleotide (FMN) and flavin adenine dinucleotide (FAD), serve as crucial cofactors for a variety of enzyme-catalyzed reactions [36]. Severe dietary deficiency of B2 can impair mitochondrial function and result in both motor and sensory PN, as well as cranial nerve involvement associated with optic atrophy [10].

Causes of riboflavin deficiency include congenital genetic disorders, malnutrition, and various environmental and physiological stressors, such as infections, exercise, diet, ageing and pregnancy [36]. Notably, suboptimal riboflavin status has been observed in approximately 10% of both omnivores and vegetarians, and in over 30% of vegans, likely due to dietary limitation [37].

To summarize, peripheral neuropathies are a common complication of B-vitamin deficiencies, particularly in high-risk groups, such as individuals with chronic alcohol use, anorexia, gastritis, or those who have undergone bariatric or metabolic surgery. The prevalence of B-vitamin-related neuropathies is expected to rise with the increasing popularity of vegan diets and in ageing populations. In addition to lifestyle and dietary factors, genetic predispositions may also contribute to an individual’s susceptibility to B-vitamin deficiencies and their neurological consequences. Routine blood tests often lack specificity, and standard measurements of total vitamin B1, B2, B6, and B12 levels do not reliably reflect functional status. More accurate assessment frequently requires evaluation of downstream metabolic markers such as homocysteine (relevant to several B vitamins) or methylmalonic acid (MMA) in cases of functional B12 deficiency (Table 1).

There is a clear need for further research into specific and sensitive biomarkers for diagnosing B-vitamin deficiencies and their association or correlation with PN.

However, the clinical priority remains early identification of individuals at-risk and the prevention of deficiencies through appropriate B-vitamin supplementation.

**Table 1:** Biomarkers of vitamin B-Status

Vitamin	Material	Parameter
**B12**	EDTA (whole blood)	Low: Hb, Ery High: MCV, MCH
	Serum/Lithiumheparin plasma	Low: total B12 (Cobalamin)
	Serum	Low: Holotranscobalamin (holoTC, active B12)
	Serum/Urine	High: Methylmalonic acid (MMA)
	Serum/Lithiumheparin plasma	High: Homocysteine(Hcy)
**B9**	EDTA (whole blood) Serum/Lithiumheparin plasma Serum/Lithiumheparin plasma	Low: Hb, Ery High: MCH, MCV Low: Folate High: Homcysteine (Hcy)
**B1**	EDTA (whole blood) Serum/Lithiumheparin plasma	Low: Hb, B1 High: Homocysteine (Hcy)
**B6**	EDTA (whole blood)	Low: Hb, MCH, MCV, B6
**B2**	EDTA (whole blood, light-protected) Serum/Lithiumheparin plasma	Low: B2 High: Homocysteine (Hcy)

Ery, erythrocyte; Hb, hemoglobin; MCH, mean corpuscular hemoglobin; MCV, mean corpuscular volume

### PN in prediabetes, diabetes and obesity

The prevalence of diabetes and prediabetes has continued to increase worldwide during recent years. The number of people living with diabetes rose from 200 million in 1990 to 828 million in 2022, of whom over 95% had type 2 diabetes (T2D) [38].

Diabetes mellitus represents the most common cause of PN, with a prevalence of up to 50% in various clinical studies along with retinopathy, nephropathy, and vasculopathy [1]. Its prevalence seems to correlate with disease duration and glycemic control [4]. The most common and typical form of diabetic neuropathy (DN) is a distal symmetric sensorimotor and autonomic neuropathy, accounting for approximately 90% of cases [39].

Autonomic neuropathy as a serious complication of diabetes is an independent predictor of cardiovascular morbidity and mortality in patients with type 1 diabetes (T1D) and T2D diabetes [40].

In addition to duration of diabetes, the prevalence also appears to be associated with other risk factors, such as obesity and the metabolic syndrome [41, 42]. Importantly, we have previously demonstrated that children and adolescents with obesity and normal glucose tolerance show autonomic nervous system dysfunction affecting several organs with a disordered activity of parasympathetic and sympathetic systems, suggesting that obesity may lead to a PN phenotype similar to that observed in children and adolescents with diabetes [43]. Furthermore, there is an increased risk for PN (distal sensorimotor PN and cardiovascular autonomic neuropathy) in people with prediabetes [44]. In both prediabetes and newly diagnosed T2D, additional risk factors for early PN were age, HbA1c, BMI, total cholesterol, albuminuria, and hypertension [45]. Interestingly, the significance of an adipocyte-PN link has been recently demonstrated in animal experiments. Thus, Schwann cells are sensitive to the key adipocytokine leptin after peripheral nerve injuries. In more detail, leptin supports functional nerve regeneration, which was confirmed by electrophysiological studies and gait parameters, supporting the link between metabolic diseases and PN [46].

Despite intensive research in recent years, there is still no unifying concept of the pathophysiology of DN nor are satisfactory pathogenesis-oriented treatments available. DN is a multifactorial condition involving oxidative stress, mitochondrial dysfunction, neuronal apoptosis, and neuroinflammation as central pathogenic mechanisms [47]. Most mechanistic insights are derived from animal models, which have provided valuable data on inflammatory processes in peripheral nerves [47]. In animal experiments, we found evidence of pro-inflammatory changes in the sciatic nerve both in hyperglycemic mouse models of mild and severe T2D (leptin-deficient *ob/ob* mice, leptin receptor-deficient *db/db mice*), and in a non-hyperglycemic rat model of metabolic syndrome (MetS) (Wistar Ottawa Karlsburg W (RT1u) WOKW rat). Interestingly, macrophage infiltration in the peripheral nerves of animals with T2D correlated with PN, whereas in animals with MetS, this process did not result in neuropathic changes [48]. We hypothesize that autophagy may play an important role in triggering an anti-inflammatory response that protects against neuropathy, and this protective effect is associated with increased mRNA expression of anti-inflammatory cytokines [48, 49]. Furthermore, dietary intake of non-heme iron appears to be a critical factor in the pathogenesis of experimental diabetic neuropathy and neuropathy in metabolic syndrome without overt diabetes. Thus, low iron intake has been shown to trigger increased inflammatory activity in the peripheral nerves [50–52]. Moreover, different miRNAs may influence multiple DN pathways, making them promising therapeutic targets that could enable novel causal therapeutic approaches [47].

Clinical studies have found that systemic biomarkers of inflammation (i.e. higher interleukin 6 [IL-6] and tumor necrosis factor [TNF]-α levels) are associated with the onset and progression of neuropathy over 6.5 years in elderly individuals with diabetes [53]. An association between distinct inflammatory biomarkers and reduced autonomic cardiac function in T2D and T1D has also been demonstrated [53–55]. However, there is currently insufficient evidence to support the relevance of screening individual inflammatory biomarker in patients [55].

Interestingly, diabetes mellitus is currently classified into four major types: T1D, T2D, other specific types, and gestational diabetes. However, especially within T2D, substantial heterogeneity has been observed in recent years. Ahlqvist et al. conducted a data-driven cluster analysis and identified five replicable subgroups of diabetes patients, each with distinct clinical characteristics and risks for complications [56]. Notably, individuals in the severe insulin-resistant diabetes (SIRD) cluster exhibited a significantly higher risk of diabetic kidney disease compared to those in clusters, such as mild obesity-related diabetes (MOD) and mild age-related diabetes (MARD) [56].

Given that microvascular complications, including nephropathy, retinopathy, and neuropathy, and others, often co-occur [57, 58], a differential risk for PN across these subgroups is plausible. Recent studies have validated this hypothesis, showing that patients with T2D and high insulin resistance [57, 59] and/or hyperinsulinemia [60] are at increased risk for PN. Thus, in a large Danish cohort, hyperinsulinemic patients had a 23% prevalence of DN, significantly higher than other subgroups, even after adjusting for metabolic syndrome components [60].

Supporting this, the LIFE-Adult Study in Germany demonstrated that markers of insulin resistance (e.g., HOMA2-IR) were independently associated with thinning of retinal layers, particularly the outer nuclear layer and myoid zone of photoreceptors [61]. These findings suggest a broader link between insulin resistance and neuronal structural changes, potentially extending to peripheral nerves. Future studies, therefore, need to investigate whether lowering of insulin resistance, e.g. through novel incretine-based drugs, can mitigate microvascular and particularly PN risk in patients with T2D. However, it is important to note that insulin resistance may not predict progression of microvascular diseases once they are already established. In the FIDELITY dataset [62], surrogate markers of insulin resistance (e.g., estimated glucose disposal rate, eGDR) were not longitudinally associated with adverse renal outcomes in patients with T2D and chronic kidney disease. This underscores the need for further research to determine whether insulin resistance similarly affects the progression of PN in patients with an already existing DN diagnosis.

Treatment-induced neuropathy in diabetes (TIND) is a rare but clinically significant subtype of diabetic neuropathy that occurs following rapid improvement in glycemic control. It affects up to 10% of patients with diabetic neuropathy undergoing intensive glucose-lowering therapy, irrespective of treatment modality, particularly when HbA1c levels drop by more than 2–3% points within a three-month period [63]. The pathophysiology of TIND remains incompletely understood. However, current hypotheses suggest that abrupt glycemic normalization may trigger a cascade of metabolic and inflammatory responses, including the release of pro-inflammatory cytokines and impaired axonal transport [63]. These changes predominantly affect small nerve fibers, leading to neuropathic pain as the primary clinical manifestation. Autonomic dysfunction, retinopathy, and nephropathy frequently co-occur, indicating a broader microvascular involvement [63].

Standard electrophysiological tests often fail to detect abnormalities in TIND due to the selective involvement of small fibers. Therefore, specialized sensory and autonomic testing, such as cardiovascular autonomic reflex tests, pupillography, thermography, and sudomotor axon reflex testing, is required to identify functional impairments [64]. A prospective pilot study by our group investigated functional predictors of TIND in patients with diabetes and an HbA1c > 8.5%. Although trends toward autonomic dysfunction were observed in patients with rapid HbA1c reduction, statistical significance was not reached due to limited sample size and adherence [64]. Nonetheless, the study supports the notion that rapid glycemic correction may transiently impair autonomic function, which may later recover with stabilized glucose levels.

In our previous study, we developed a T1D rat model of TIND [65]. This model successfully reproduced key clinical features observed in humans, including the rapid onset of neuropathic symptoms following intensive insulin therapy. Our T1D-TIND model (bio breeding/Ottawa Karlsburg Leipzig diabetic rats) provides a valuable preclinical platform to further explore of the underlying mechanisms and supports the development of future diagnostic and therapeutic approaches for affected patients.

Given the lack of specific treatment guidelines, supportive care remains the mainstay of TIND management. Gradual glycemic control may help prevent its onset, although prospective trials are needed to validate this approach.

### Treatment and prevention

Nutritional and metabolic risk factors play an important role in the development and progression of PN. Important causes include vitamin B deficiency and diabetes, as well as a pro-inflammatory milieu. Therefore, patients at risk should be identified and screened for vitamin B deficiency. Risk factors for vitamin B deficiency include alcohol abuse, gastrointestinal diseases with malabsorption or surgery, unbalanced diets (including vegan and vegetarian diets), and medications, such as antacids, metformin, levodopa/carbidopa, sodium valproate, folate antagonists, antitubercular drugs, and penicillamine. Another risk is increased need for B-vitamins, which can be observed in pregnancy, in hematological diseases, critically ill patients, or patients receiving intensive medical care (Table 2).

**Table 2:** Risk factors for nutritional and metabolic PN

Risk Factors	Predisposition/Reason
Vitamin B12 (C*obalamin)* deficiency	- Gastrointestinal and bariatric/metabolic surgery - Malabsorptive diseases (e.g. gastritis, Celiac disease, Crohn’s disease) - Medication: antacids, metformin, L-Dopa - Diet: vegetarians, vegans - Genetic factors - Increased consumption: pregnancy, breastfeeding mothers, hematological disorders - Age
Vitamin B9 *(Folate)* deficiency	- Malabsorptive diseases - Chronic alcoholism - Medication: sodium valproate, methotrexate - Increased consumption: pregnancy, hematological disorders - Genetic disorders of folate metabolism
Vitamin B1 *(Thiamin)* deficiency	- Chronic alcoholism - Gastrointestinal and bariatric surgery - Malabsorptive diseases - Breastfed infants of thiamine-deficient mothers - Monotonous diet with starchy, thiamine-poor staple foods - Thiamine antagonists: betel nuts or tea - Increased consumption: critically ill patients or patients receiving intensive medical care - Intensive physical exercise, fiber, high stress
Vitamin B6 *(Pyridoxine)* deficiency	- Severe malnutrition - Alcohol excesses - Diabetes - Medication: antituberculosis drug isoniazine, hydralazine, penicillamine
Vitamin B2 *(Riboflavin)* deficiency	- Malnutrition - Diet: vegetarians, vegans - Increased consumption: exercise, ageing, pregnancy
Prediabetes	- Age - BMI - Total cholesterol - Hypertension - Inflammation
Diabetes	- HbA1c - Duration of diabetes - Age - Inflammation - Rapid reduction of HbA1c under therapy (TIND)
Obesity and Metabolic Syndrome	- Age - Prediabetes - Dyslipidemia - Inflammation

BMI, body mass index; HbA1c, hemoglobin A1c; L-Dopa, Levodopa (L-3, 4-dihydroxyphenylalanine); TIND, treatment-induced neuropathy of diabetes

In addition to identifying patients at risk, the prevention of B vitamin deficiency is important to avoid PN. B12 deficiency is treated orally or, if necessary, parenterally depending on the underlying cause. In addition to active absorption, approximately 1–2% of free vitamin B12 can be absorbed via passive diffusion. Therefore, high-dose oral administration can also be effective (e.g., in patients with T2D on metformin). Thus, in patients with T2D, treatment with 1 mg oral methylcobalamin for twelve months increased plasma B12 levels and partially improved neurophysiological parameters, pain scores, and quality of life [66].

It is important to continue monitoring vitamin B12 status following supplementation to ensure adequate replacement [10]. More attention must be paid to vitamin B12 in a strict vegan and vegetarian diet, especially regarding elevated homocysteine levels in plasma. If homocysteine levels are elevated, the other B vitamins should also be checked. Only pyridoxine (B6) status appears to be independent of the diet [37].

Other important risk factors for metabolic PN are obesity, metabolic syndrome prediabetes, and T1D and T2D (Table 2). People living with overweight or obesity should be screened for diabetes. Several studies have shown that lifestyle changes can prevent T2D in overweight people with prediabetes [67]. We observed stabilization and improvement of autonomic neuropathy in correlation with improvements in body weight status and metabolic risk factors after a multimodal lifestyle intervention, i.e. a combination of physical exercise, nutritional counseling, and psychological support, even in childhood obesity [68].

A cross-sectional study showed that higher physical activity, a healthy diet, low body mass index (BMI), non-smoking status, lower total cholesterol and hemoglobin A1c (HbA1c), as well as blood pressure, were associated with a lower incidence of PN in US adults, regardless of diabetes status [69]. Mechanistically, physical exercise could improve the pro-inflammatory status in patients with T2D and/or obesity [70]. However, as IL-6 is also regarded as a potentially beneficial myokine which is increased during physical exercise [71, 72], other effects might also be causally involved.

In general, intensive blood glucose control reduced the risk of developing PN in patients with T1D and cardiovascular and mortality risk associated with cardiac autonomic neuropathy in patients with T2D. Intensive glucose control, therefore, is an crucial and effective element of treatment [73, 74]. However, especially in patients with very high HbA1c, there is a high risk for TIND and early treatment intensity and glucose control need to be balanced with TIND risk. Furthermore, education of primary care physicians, Neurologists, and Endocrinologists is essential to prevent TIND in patients with diabetes.

Currently, there are no effective therapeutic strategies for diabetic neuropathy, primarily due to the lack of treatments that directly target the underlying nerve damage. While early and intensive blood glucose control has been shown to reduce the incidence of neuropathy in patients with T1D, its effectiveness in T2D is limited. As mentioned above, the inflammatory response plays a crucial role in the pathogenesis of diabetic neuropathy [75]. In diabetic conditions, increased oxidative stress and generation of reactive oxygen species (ROS) contribute to macrophage recruitment and production of pro-inflammatory cytokines, which in turn is associated with neuropathic pain [76, 77]. Therefore, targeting inflammation and oxidative stress represents a promising therapeutic and preventive approach for this disease.

Given that metabolic PN is a multifactorial disease, effective prevention and treatment strategies must be multidimensional. Preventive measures should involve several steps, beginning with active prevention through the identification of risk factors and early intervention. These include lifestyle modifications, such as regular physical activity, weight control, adherence to a healthy diet, supplementation with B vitamins when indicated, smoking cessation, and reduction of alcohol consumption. Moreover, anti‑inflammatory and antioxidant supplementation, for instance, nicotinamide mononucleotide (NMN) and omega‑3 fatty acids, may provide additional benefits. In clinical trials, NMN has been shown to improve insulin sensitivity and physical performance in prediabetic individuals [78]. NMN’s anti-inflammatory, anti-oxidative, and anti-aging properties suggest potential benefits for nerve function in metabolic neuropathies [79].

Moreover, anti-inflammatory and antioxidative supplementation, such as omega-3 fatty acids, has been suggested to modulate neuroinflammation and oxidative stress in patients with neuropathic conditions [80].

**Fig. 1:**
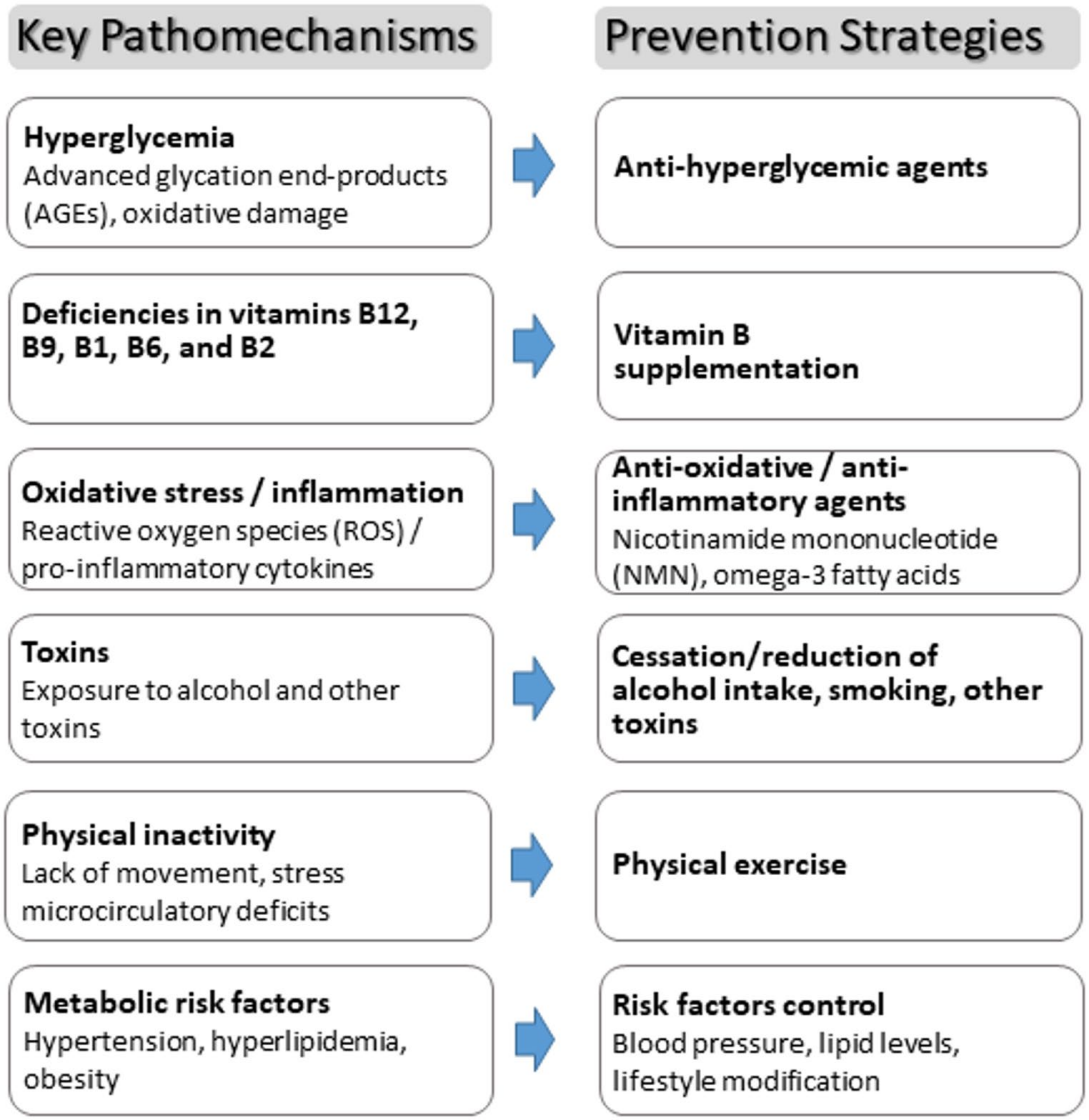
Pathomechanisms and prevention of peripheral neuropathy (PN). Overview on key pathomechanisms and potential prevention strategies for the development and progression of PN

In addition, a shift toward pathomechanism-based therapeutic approaches is essential to address the underlying pathophysiological processes (Fig. 1). A comprehensive, individualized approach that combines these interventions is crucial for effectively preventing the onset and progression of metabolic polyneuropathies.

## Conclusion

In summary, awareness of neuropathies in individuals with metabolic diseases or predispositions listed in this article, such as conditions leading to vitamin deficiencies, prediabetes, obesity and diabetes as key risk factors, needs to be increased.

Current knowledge of these conditions should be translated into clinical practice and public health initiatives. Beyond preventive measures, further research is required on therapeutic agents that target these diseases causally through different signaling pathways. It is interesting to note in this context that there is a lack of evidence from randomized controlled trials specific to PN, in contrast to other microvascular diseases, such as diabetic kidney disease, and that there is an urgent need for more clinical research on PN.

## Data Availability

No new data were generated or analyzed in this study. The data used and/or analyzed are available from the cited sources and corresponding authors upon reasonable request.

## Abbreviations

5-MTHF: 5-methyltetrahydrofolate
AGEs: Advanced glycation end-products
BMI: Body mass index
DNA: Deoxyribonucleic acid
DN: Diabetic neuropathy
FAD: Flavin adenine dinucleotide
FIDELITY: Finerenone in diabetes and kidney disease study
FMN: Flavin mononucleotide
HbA1c: Hemoglobin A1c
Hcy: Homocysteine
holoTC: Holotranscobalamin
HOMA2-IR: Homeostasis model assessment of insulin resistance (version 2)
IL-6: Interleukin 6
KORA: Cooperative health research in the augsburg region
L-Dopa: Levodopa (L-3,4-dihydroxyphenylalanine)
MARD: Mild age-related diabetes
MMA: Methylmalonic acid
MetS: Metabolic syndrome
MOD: Mild obesity-related diabetes
MTHFR: 5,10-methylenetetrahydrofolate reductase
NMN: Nicotinamide mononucleotide
RNA: Ribonucleic acid
ROS: Reactive oxygen species
T1D: Type 1 diabetes
T2D: Type 2 diabetes
TIND: Treatment-induced neuropathy of diabetes
TNF-α: Tumor necrosis factor alpha
WOKW: Wistar Ottawa Karlsburg W (RT1u)

## References

[1] Hanewinckel, R., van Oijen, M., Ikram, M. A., & van Doorn, P. A. (2016). The epidemiology and risk factors of chronic polyneuropathy. *European Journal of Epidemiology,**31*, 5–20. 10.1007/s10654-015-0094-6 26700499 10.1007/s10654-015-0094-6 PMC4756033

[2] Hicks, C. W., Wang, D., Windham, B. G., Matsushita, K., & Selvin, E. (2021). Prevalence of peripheral neuropathy defined by monofilament insensitivity in middle-aged and older adults in two US cohorts. *Scientific Reports,**11*, Article 19159. 10.1038/s41598-021-98565-w 34580377 10.1038/s41598-021-98565-w PMC8476511

[3] Hoffman, E. M., Staff, N. P., Robb, J. M., St Sauver, J. L., Dyck, P. J., & Klein, C. J. (2015). Impairments and comorbidities of polyneuropathy revealed by population-based analyses. *Neurology,**84*, 1644–51. 10.1212/WNL.0000000000001492 25832668 10.1212/WNL.0000000000001492 PMC4409579

[4] Tesfaye, S., & Selvarajah, D. (2012). Advances in the epidemiology, pathogenesis and management of diabetic peripheral neuropathy. *Diabetes/Metabolism Reviews*, *28*(Suppl 1), 8–14. 10.1002/dmrr.2239 10.1002/dmrr.2239 22271716

[5] Hanewinckel, R., Drenthen, J., van Oijen, M., Hofman, A., van Doorn, P. A., & Ikram, M. A. (2016). Prevalence of polyneuropathy in the general middle-aged and elderly population. *Neurology,**87*, 1892–8. 10.1212/WNL.0000000000003293 27683845 10.1212/WNL.0000000000003293

[6] Taams, N. E., Drenthen, J., Hanewinckel, R., Ikram, M. A., & van Doorn, P. A. (2022). Prevalence and risk factor profiles for chronic axonal polyneuropathy in the general population. *Neurology,**99*, e2234–e2240. 10.1212/WNL.0000000000201168 36008153 10.1212/WNL.0000000000201168

[7] Hamel, J. I., & Logigian, E. L. (2023). Clinical spectrum and prognosis in patients with acute nutritional axonal neuropathy. *Neurology*, *100*, e2134–e2140. 10.1212/WNL.0000000000207215 36973043 10.1212/WNL.0000000000207215 PMC10186231

[8] Solomon, L. R. (2007). Disorders of cobalamin (vitamin B12) metabolism: Emerging concepts in pathophysiology, diagnosis and treatment. *Blood Reviews,**21*, 113–30. 10.1016/j.blre.2006.05.001 16814909 10.1016/j.blre.2006.05.001

[9] Solomon, L. R. (2016). Vitamin B12-responsive neuropathies: A case series. *Nutritional Neuroscience,**19*, 162–8. 10.1179/1476830515Y.0000000006 25710280 10.1179/1476830515Y.0000000006

[10] Kramarz, C., Murphy, E., Reilly, M. M., & Rossor, A. M. (2023). Nutritional peripheral neuropathies. *Journal of Neurology, Neurosurgery & Psychiatry,**95*, 61–72. 10.1136/jnnp-2022-329849 37536924 10.1136/jnnp-2022-329849

[11] Harrington, D. J., Stevenson, E., & Sobczyńska-Malefora, A. (2025). The application and interpretation of laboratory biomarkers for the evaluation of vitamin B12 status. *Annals of Clinical Biochemistry,**62*, 22–33. 10.1177/00045632241292432 39367523 10.1177/00045632241292432 PMC11707970

[12] Arnold, T., & Johnston, C. S. (2023). An examination of relationships between vitamin B12 status and functional measures of peripheral neuropathy in young adult vegetarians. *Frontiers in Nutrition,**10*, Article 1304134. 10.3389/fnut.2023.1304134 38174111 10.3389/fnut.2023.1304134 PMC10764020

[13] Leishear, K., Boudreau, R. M., Studenski, S. A., Ferrucci, L., Rosano, C., de Rekeneire, N., et al. (2012). Relationship between vitamin B12 and sensory and motor peripheral nerve function in older adults. *Journal of the American Geriatrics Society*, *60*, 1057–1063. 10.1111/j.1532-5415.2012.03998.x 22690982 10.1111/j.1532-5415.2012.03998.x PMC3376015

[14] Warendorf, J. K., van Doormaal, P. T. C., Vrancken, A. F. J. E., Verhoeven-Duif, N. M., van Eijk, R. P. A., van den Berg, L. H., & Notermans, N. C. (2022). Clinical relevance of testing for metabolic vitamin B12 deficiency in patients with polyneuropathy. *Nutritional Neuroscience,**25*, 2536–46. 10.1080/1028415X.2021.1985751 34693890 10.1080/1028415X.2021.1985751

[15] Battat, R., Kopylov, U., Szilagyi, A., Saxena, A., Rosenblatt, D. S., Warner, M., et al. (2014). Vitamin B12 deficiency in inflammatory bowel disease: Prevalence, risk factors, evaluation, and management. *Inflammatory Bowel Diseases,**20*, 1120–8. 10.1097/MIB.0000000000000024 24739632 10.1097/MIB.0000000000000024

[16] Bell, D. S. H. (2022). Metformin-induced vitamin B12 deficiency can cause or worsen distal symmetrical, autonomic and cardiac neuropathy in the patient with diabetes. *Diabetes, Obesity & Metabolism,**24*, 1423–8. 10.1111/dom.14734 10.1111/dom.14734 35491956

[17] Schiavon, C. A., Bhatt, D. L., Ikeoka, D., Santucci, E. V., Santos, R. N., Damiani, L. P., et al. (2020). Three-year outcomes of bariatric surgery in patients with obesity and hypertension: A randomized clinical trial. *Annals of Internal Medicine,**173*, 685–93. 10.7326/M19-3781 32805133 10.7326/M19-3781

[18] Jung, S. B., Nagaraja, V., Kapur, A., & Eslick, G. D. (2015). Association between vitamin B12 deficiency and long-term use of acid-lowering agents: A systematic review and meta-analysis. *Internal Medicine Journal,**45*, 409–16. 10.1111/imj.12697 25583062 10.1111/imj.12697

[19] Gilsing, A. M. J., Crowe, F. L., Lloyd-Wright, Z., Sanders, T. A. B., Appleby, P. N., Allen, N. E., & Key, T. J. (2010). Serum concentrations of vitamin B12 and folate in British male omnivores, vegetarians and vegans: Results from a cross-sectional analysis of the EPIC-Oxford cohort study. *European Journal of Clinical Nutrition,**64*, 933–9. 10.1038/ejcn.2010.142 20648045 10.1038/ejcn.2010.142 PMC2933506

[20] Yang, W., Cai, X., Wu, H., & Ji, L. (2019). Associations between metformin use and vitamin B12 levels, anemia, and neuropathy in patients with diabetes: A meta-analysis. *Journal of Diabetes,**11*, 729–743. 10.1111/1753-0407.12900 30615306 10.1111/1753-0407.12900

[21] Rekik, A., Santoro, C., Poplawska-Domaszewicz, K., Qamar, M. A., Batzu, L., Landolfo, S., et al. (2024). Parkinson’s disease and vitamins: A focus on vitamin B12. *Journal of Neural Transmission,**131*, 1495–509. 10.1007/s00702-024-02769-z 38602571 10.1007/s00702-024-02769-z PMC11608379

[22] Kwon, E. H., Steininger, J., Scherbaum, R., Gold, R., Pitarokoili, K., & Tönges, L. (2024). Large-fiber neuropathy in Parkinson’s disease: A narrative review. *Neurology Research and Practice,**6*, Article 51. 10.1186/s42466-024-00354-z 10.1186/s42466-024-00354-z PMC11514528 39465424

[23] Conzade, R., Koenig, W., Heier, M., Schneider, A., Grill, E., Peters, A., & Thorand, B. (2017). Prevalence and predictors of subclinical micronutrient deficiency in German older adults: Results from the Population-Based KORA-Age study. *Nutrients*. 10.3390/nu9121276 29168737 10.3390/nu9121276 PMC5748727

[24] Niklewicz, A., Hannibal, L., Warren, M., & Ahmadi, K. R. (2024). A systematic review and meta-analysis of functional vitamin B12 status among adult vegans. *Nutrition Bulletin,**49*, 463–479. 10.1111/nbu.12712 39373282 10.1111/nbu.12712

[25] Gallego-Narbón, A., Zapatera, B., Barrios, L., & Vaquero, M. P. (2019). Vitamin B12 and folate status in Spanish lacto-ovo vegetarians and vegans. *Journal of Nutritional Science,**8*, Article e7. 10.1017/jns.2019.2 30828450 10.1017/jns.2019.2 PMC6391582

[26] Storz, M. A., Müller, A., Niederreiter, L., Zimmermann-Klemd, A. M., Suarez-Alvarez, M., Kowarschik, S., et al. (2023). A cross-sectional study of nutritional status in healthy, young, physically-active German omnivores, vegetarians and vegans reveals adequate vitamin B12 status in supplemented vegans. *Annals of Medicine,**55*, Article 2269969. 10.1080/07853890.2023.2269969 37851870 10.1080/07853890.2023.2269969 PMC10586079

[27] Taverner, T., Crowe, F. L., Thomas, G. N., Gokhale, K., Thayakaran, R., Nirantharakumar, K., & Rajabally, Y. A. (2019). Circulating folate concentrations and risk of peripheral neuropathy and mortality: A retrospective cohort study in the U.K. *Nutrients*. 10.3390/nu11102443 31614995 10.3390/nu11102443 PMC6835340

[28] Wilcken, B., Bamforth, F., Li, Z., Zhu, H., Ritvanen, A., Renlund, M., et al. (2003). Geographical and ethnic variation of the 677CT allele of 5,10 methylenetetrahydrofolate reductase (MTHFR): Findings from over 7000 newborns from 16 areas world wide. *Journal of Medical Genetics*, *40*, 619–625. 10.1136/jmg.40.8.619 12920077 10.1136/jmg.40.8.619 PMC1735571

[29] Prinz-Langenohl, R., Brämswig, S., Tobolski, O., Smulders, Y. M., Smith, D. E. C., Finglas, P. M., & Pietrzik, K. (2009). 6S-5-methyltetrahydrofolate increases plasma folate more effectively than folic acid in women with the homozygous or wild-type 677C–T polymorphism of methylenetetrahydrofolate reductase. *British Journal of Pharmacology,**158*, 2014–21. 10.1111/j.1476-5381.2009.00492.x 19917061 10.1111/j.1476-5381.2009.00492.x PMC2807663

[30] Koike, H., Iijima, M., Sugiura, M., Mori, K., Hattori, N., Ito, H., et al. (2003). Alcoholic neuropathy is clinicopathologically distinct from thiamine-deficiency neuropathy. *Annals of Neurology,**54*, 19–29. 10.1002/ana.10550 12838517 10.1002/ana.10550

[31] Whitfield, K. C., Bourassa, M. W., Adamolekun, B., Bergeron, G., Bettendorff, L., Brown, K. H., et al. (2018). Thiamine deficiency disorders: Diagnosis, prevalence, and a roadmap for global control programs. *Annals of the New York Academy of Sciences,**1430*, 3–43. 10.1111/nyas.13919 30151974 10.1111/nyas.13919 PMC6392124

[32] Whitfield, K. C., Smith, G., Chamnan, C., Karakochuk, C. D., Sophonneary, P., Kuong, K., et al. (2017). High prevalence of thiamine (vitamin B1) deficiency in early childhood among a nationally representative sample of Cambodian women of childbearing age and their children. *PLoS Neglected Tropical Diseases,**11*, Article e0005814. 10.1371/journal.pntd.0005814 28873391 10.1371/journal.pntd.0005814 PMC5600402

[33] Ghavanini, A. A., & Kimpinski, K. (2014). Revisiting the evidence for neuropathy caused by pyridoxine deficiency and excess. *Journal of Clinical Neuromuscular Disease,**16*, 25–31. 10.1097/CND.0000000000000049 25137514 10.1097/CND.0000000000000049

[34] Vrolijk, M. F., Opperhuizen, A., Jansen, E. H. J. M., Hageman, G. J., Bast, A., & Haenen, G. R. M. M. (2017). The vitamin B6 paradox: Supplementation with high concentrations of pyridoxine leads to decreased vitamin B6 function. *Toxicology In Vitro,**44*, 206–12. 10.1016/j.tiv.2017.07.009 28716455 10.1016/j.tiv.2017.07.009

[35] Muhamad, R., Akrivaki, A., Papagiannopoulou, G., Zavridis, P., & Zis, P. (2023). The role of vitamin B6 in peripheral neuropathy: A systematic review. *Nutrients*. 10.3390/nu15132823 37447150 10.3390/nu15132823 PMC10343656

[36] Mosegaard, S., Dipace, G., Bross, P., Carlsen, J., Gregersen, N., & Olsen, R. K. J. (2020). Riboflavin deficiency-implications for general human health and inborn errors of metabolism. *International Journal of Molecular Sciences*. 10.3390/ijms21113847 32481712 10.3390/ijms21113847 PMC7312377

[37] Majchrzak, D., Singer, I., Männer, M., Rust, P., Genser, D., Wagner, K-H., & Elmadfa, I. (2006). B-vitamin status and concentrations of homocysteine in Austrian omnivores, vegetarians and vegans. *Annals of Nutrition & Metabolism*, *50*, 485–491. 10.1159/000095828 16988496 10.1159/000095828

[38] Worldwide trends in. (2024). Diabetes prevalence and treatment from 1990 to 2022: A pooled analysis of 1108 population-representative studies with 141 million participants. *Lancet*, *404*, 2077–2093. 10.1016/S0140-6736(24)02317-1 39549716 10.1016/S0140-6736(24)02317-1 PMC7616842

[39] Albers, J. W., & Pop-Busui, R. (2014). Diabetic neuropathy: Mechanisms, emerging treatments, and subtypes. *Current Neurology and Neuroscience Reports,**14*, Article 473. 10.1007/s11910-014-0473-5 24954624 10.1007/s11910-014-0473-5 PMC5084622

[40] Duque, A., Mediano, M. F. F., Lorenzo, A., & Rodrigues, L. F. (2021). Cardiovascular autonomic neuropathy in diabetes: Pathophysiology, clinical assessment and implications. *World Journal of Diabetes,**12*, 855–67. 10.4239/wjd.v12.i6.855 34168733 10.4239/wjd.v12.i6.855 PMC8192252

[41] Andersen, S. T., Witte, D. R., Dalsgaard, E.-M., Andersen, H., Nawroth, P., Fleming, T., et al. (2018). Risk factors for incident diabetic polyneuropathy in a cohort with screen-detected type 2 diabetes followed for 13 years: ADDITION-Denmark. *Diabetes Care,**41*, 1068–75. 10.2337/dc17-2062 29487078 10.2337/dc17-2062

[42] Callaghan, B. C., Gao, L., Li, Y., Zhou, X., Reynolds, E., Banerjee, M., et al. (2018). Diabetes and obesity are the main metabolic drivers of peripheral neuropathy. *Annals of Clinical and Translational Neurology,**5*, 397–405. 10.1002/acn3.531 29687018 10.1002/acn3.531 PMC5899909

[43] Baum, P., Petroff, D., Classen, J., Kiess, W., & Blüher, S. (2013). Dysfunction of autonomic nervous system in childhood obesity: A cross-sectional study. *PLoS ONE,**8*, Article e54546. 10.1371/journal.pone.0054546 23358101 10.1371/journal.pone.0054546 PMC3554723

[44] Ziegler, D., Herder, C., & Papanas, N. (2023). Neuropathy in prediabetes. *Diabetes, Metabolic Research and Reviews,**39*, Article e3693. 10.1002/dmrr.3693 10.1002/dmrr.3693 37470302

[45] Mairaj, M. K., Pala, N. A., & Ismail, M. (2024). Profiles of peripheral neuropathy and risk factors in people with treatment-naive type 2 diabetes mellitus and prediabetes. *Cureus,**16*, Article e73304. 10.7759/cureus.73304 39655103 10.7759/cureus.73304 PMC11625967

[46] Sundaram, V. K., Schütza, V., Schröter, N. H., Backhaus, A., Bilsing, A., Joneck, L., et al. (2023). Adipo-glial signaling mediates metabolic adaptation in peripheral nerve regeneration. *Cell Metab*, *35*, 2136–2152e9. 10.1016/j.cmet.2023.10.017 37989315 10.1016/j.cmet.2023.10.017 PMC10722468

[47] Panou, T., Gouveri, E., Popovic, D. S., Papazoglou, D., & Papanas, N. (2025). The role of inflammation in the pathogenesis of diabetic peripheral neuropathy: New lessons from experimental studies and clinical implications. *Diabetes Therapy,**16*, 371–411. 10.1007/s13300-025-01699-7 39928224 10.1007/s13300-025-01699-7 PMC11868477

[48] Kosacka, J., Nowicki, M., Blüher, M., Baum, P., Stockinger, M., Toyka, K. V., et al. (2013). Increased autophagy in peripheral nerves may protect Wistar Ottawa Karlsburg W rats against neuropathy. *Experimental Neurology,**250*, 125–35. 10.1016/j.expneurol.2013.09.017 24095727 10.1016/j.expneurol.2013.09.017

[49] Baum, P., Ebert, T., Klöting, N., Krupka, S., König, M., Paeschke, S., et al. (2025). Inflammation and autophagy in peripheral nerves of rodent models with metabolic syndrome and type 2 diabetes mellitus. *Neuroscience Research*. 10.1016/j.neures.2025.04.002 40252698 10.1016/j.neures.2025.04.002

[50] Baum, P., Kosacka, J., Estrela-Lopis, I., Woidt, K., Serke, H., Paeschke, S., et al. (2016). The role of nerve inflammation and exogenous iron load in experimental peripheral diabetic neuropathy (PDN). *Metabolism,**65*, 391–405. 10.1016/j.metabol.2015.11.002 26975531 10.1016/j.metabol.2015.11.002

[51] Kosacka, J., Woidt, K., Toyka, K. V., Paeschke, S., Kloting, N., Bechmann, I., et al. (2019). The role of dietary non-heme iron load and peripheral nerve inflammation in the development of peripheral neuropathy (PN) in obese non-diabetic leptin-deficient ob/ob mice. *Neurological Research*. 10.1080/01616412.2018.1564191 30638160 10.1080/01616412.2018.1564191

[52] Paeschke, S., Baum, P., Toyka, K. V., Blüher, M., Koj, S., Klöting, N., et al. (2019). The role of iron and nerve inflammation in diabetes mellitus type 2-induced peripheral neuropathy. *Neuroscience,**406*, 496–509. 10.1016/j.neuroscience.2019.03.005 30867132 10.1016/j.neuroscience.2019.03.005

[53] Herder, C., Kannenberg, J. M., Huth, C., Carstensen-Kirberg, M., Rathmann, W., Koenig, W., et al. (2017). Proinflammatory cytokines predict the incidence and progression of distal sensorimotor polyneuropathy: KORA F4/FF4 study. *Diabetes Care,**40*, 569–76. 10.2337/dc16-2259 28174259 10.2337/dc16-2259

[54] Herder, C., Schamarek, I., Nowotny, B., Carstensen-Kirberg, M., Strassburger, K., Nowotny, P., et al. (2017). Inflammatory markers are associated with cardiac autonomic dysfunction in recent-onset type 2 diabetes. *Heart,**103*, 63–70. 10.1136/heartjnl-2015-309181 27481890 10.1136/heartjnl-2015-309181

[55] Ang, L., Gunaratnam, S., Huang, Y., Dillon, B. R., Martin, C. L., Burant, A., et al. (2025). Inflammatory markers and measures of cardiovascular autonomic neuropathy in type 1 diabetes. *Journal of the American Heart Association,**14*, Article e036787. 10.1161/JAHA.124.036787 39727210 10.1161/JAHA.124.036787 PMC12054404

[56] Ahlqvist, E., Storm, P., Käräjämäki, A., Martinell, M., Dorkhan, M., Carlsson, A., et al. (2018). Novel subgroups of adult-onset diabetes and their association with outcomes: A data-driven cluster analysis of six variables. *The Lancet Diabetes & Endocrinology,**6*, 361–9. 10.1016/S2213-8587(18)30051-2 29503172 10.1016/S2213-8587(18)30051-2

[57] Li, J., Cao, Y., Liu, W., Wang, Q., Qian, Y., & Lu, P. (2019). Correlations among diabetic microvascular complications: A systematic review and meta-analysis. *Scientific Reports,**9*, Article 3137. 10.1038/s41598-019-40049-z 30816322 10.1038/s41598-019-40049-z PMC6395813

[58] Ebert, T., Widman, L., Stenvinkel, P., & Hagström, H. (2023). Increased risk for microvascular outcomes in NAFLD-A nationwide, population-based cohort study. *Journal of Internal Medicine*, *294*, 216–227. 10.1111/joim.13673 37259481 10.1111/joim.13673

[59] Xing, L., Peng, F., Liang, Q., Dai, X., Ren, J., Wu, H., et al. (2021). Clinical characteristics and risk of diabetic complications in data-driven clusters among type 2 diabetes. *Frontiers in Endocrinology (Lausanne),**12*, Article 617628. 10.3389/fendo.2021.617628 10.3389/fendo.2021.617628 PMC8281969 34276555

[60] Kristensen, F. P. B., Christensen, D. H., Callaghan, B. C., Stidsen, J. V., Nielsen, J. S., Højlund, K., et al. (2023). The prevalence of polyneuropathy in type 2 diabetes subgroups based on HOMA2 indices of β-cell function and insulin sensitivity. *Diabetes Care,**46*, 1546–55. 10.2337/dc23-0079 37335990 10.2337/dc23-0079

[61] Rauscher, F. G., Elze, T., Francke, M., Martinez-Perez, M. E., Li, Y., Wirkner, K., et al. (2024). Glucose tolerance and insulin resistance/sensitivity associate with retinal layer characteristics: The LIFE-Adult-Study. *Diabetologia,**67*, 928–39. 10.1007/s00125-024-06093-9 38431705 10.1007/s00125-024-06093-9 PMC10954961

[62] Ebert, T., Anker, S. D., Ruilope, L. M., Fioretto, P., Fonseca, V., Umpierrez, G. E., et al. (2024). Outcomes with Finerenone in patients with chronic kidney disease and type 2 diabetes by baseline insulin resistance. *Diabetes Care,**47*, 362–70. 10.2337/dc23-1420 38151465 10.2337/dc23-1420 PMC10909685

[63] Gibbons, C. H. (2020). Treatment induced neuropathy of diabetes. *Autonomic Neuroscience,**226*, Article 102668. 10.1016/j.autneu.2020.102668 32247944 10.1016/j.autneu.2020.102668

[64] Hoffmann, Y., Toyka, K. V., Blüher, M., Classen, J., & Baum, P. (2022). Functional predictors of treatment induced diabetic neuropathy (TIND): A prospective pilot study using clinical and neurophysiological functional tests. *Diabetology & Metabolic Syndrome,**14*, Article 35. 10.1186/s13098-022-00805-0 35241138 10.1186/s13098-022-00805-0 PMC8892777

[65] Baum, P., Koj, S., Klöting, N., Blüher, M., Classen, J., Paeschke, S., et al. (2021). Treatment-induced neuropathy in diabetes (TIND)-Developing a disease model in type 1 diabetic rats. *International Journal of Molecular Sciences*. 10.3390/ijms22041571 33557206 10.3390/ijms22041571 PMC7913916

[66] Didangelos, T., Karlafti, E., Kotzakioulafi, E., Margariti, E., Giannoulaki, P., Batanis, G., et al. (2021). Vitamin B12 supplementation in diabetic neuropathy: A 1-year, randomized, double-blind, placebo-controlled trial. *Nutrients*. 10.3390/nu13020395 33513879 10.3390/nu13020395 PMC7912007

[67] Saito, T., Watanabe, M., Nishida, J., Izumi, T., Omura, M., Takagi, T., et al. (2011). Lifestyle modification and prevention of type 2 diabetes in overweight Japanese with impaired fasting glucose levels: A randomized controlled trial. *Archives of Internal Medicine*, *171*, 1352–1360. 10.1001/archinternmed.2011.275 21824948 10.1001/archinternmed.2011.275

[68] Bluher, S., Petroff, D., Keller, A., Wagner, A., Classen, J., & Baum, P. (2014). Effect of a 1-year obesity intervention (KLAKS program) on preexisting autonomic nervous dysfunction in childhood obesity. *Journal of Child Neurology*. 10.1177/0883073814555190 25406153 10.1177/0883073814555190

[69] Gu, X., Zhu, F., Gao, P., Shen, Y., & Lu, L. (2025). Association between life’s simple 7 and peripheral neuropathy among U.S. adults, a cross-sectional study. *Journal of Health, Population and Nutrition,**44*, Article 118. 10.1186/s41043-025-00864-9 40234951 10.1186/s41043-025-00864-9 PMC11998258

[70] Hejazi, K., Mohammad Rahimi, G. R., & Rosenkranz, S. K. (2023). Effects of exercise training on inflammatory and cardiometabolic risk biomarkers in patients with type 2 diabetes mellitus: A systematic review and meta-analysis of randomized controlled trials. *Biological Research for Nursing,**25*, 250–66. 10.1177/10998004221132841 36213963 10.1177/10998004221132841

[71] Kistner, T. M., Pedersen, B. K., & Lieberman, D. E. (2022). Interleukin 6 as an energy allocator in muscle tissue. *Nature Metabolism,**4*, 170–9. 10.1038/s42255-022-00538-4 35210610 10.1038/s42255-022-00538-4

[72] Severinsen, M. C. K., & Pedersen, B. K. (2020). Muscle-organ crosstalk: The emerging roles of myokines. *Endocrine Reviews,**41*, 594–609. 10.1210/endrev/bnaa016 32393961 10.1210/endrev/bnaa016 PMC7288608

[73] Fullerton, B., Jeitler, K., Seitz, M., Horvath, K., Berghold, A., & Siebenhofer, A. (2014). Intensive glucose control versus conventional glucose control for type 1 diabetes mellitus. *Cochrane Database of Systematic Reviews,**2*, Article CD009122. 10.1002/14651858.CD009122.pub2 10.1002/14651858.CD009122.pub2 PMC6486147 24526393

[74] Zhou, H., Huang, Y., Xie, P., Zhang, S., Liu, M., Xiong, Z., et al. (2025). Intensive glycemic treatment mitigates cardiovascular and mortality risk associated with cardiac autonomic neuropathy in type 2 diabetes. *Journal of the American Heart Association,**14*, Article e035788. 10.1161/JAHA.124.035788 40314352 10.1161/JAHA.124.035788 PMC12184248

[75] Stoian, A., Muntean, C., Babă, D.-F., Manea, A., Dénes, L., Simon-Szabó, Z., et al. (2024). Update on biomarkers of chronic inflammatory processes underlying diabetic neuropathy. *International Journal of Molecular Sciences*. 10.3390/ijms251910395 39408723 10.3390/ijms251910395 PMC11476795

[76] Zamanian, M. Y., Alsaab, H. O., Golmohammadi, M., Yumashev, A., Jabba, A. M., Abid, M. K., et al. (2024). NF-κB pathway as a molecular target for curcumin in diabetes mellitus treatment: Focusing on oxidative stress and inflammation. *Cell Biochemistry and Function,**42*, Article e4030. 10.1002/cbf.4030 38720663 10.1002/cbf.4030

[77] Chong, Z. Z., & Souayah, N. (2025). Neuroinflammation in diabetic peripheral neuropathy and therapeutic implications. *Reviews in the Neurosciences*. 10.1515/revneuro-2025-0031 40228523 10.1515/revneuro-2025-0031

[78] Irie, J., Inagaki, E., Fujita, M., Nakaya, H., Mitsuishi, M., Yamaguchi, S., et al. (2020). Effect of oral administration of nicotinamide mononucleotide on clinical parameters and nicotinamide metabolite levels in healthy Japanese men. *Endocrine Journal,**67*, 153–60. 10.1507/endocrj.EJ19-0313 31685720 10.1507/endocrj.EJ19-0313

[79] Yoshino, J., Baur, J. A., & Imai, S.-I. (2018). NAD+ intermediates: The biology and therapeutic potential of NMN and NR. *Cell Metabolism,**27*, 513–528. 10.1016/j.cmet.2017.11.002 29249689 10.1016/j.cmet.2017.11.002 PMC5842119

[80] Kousparou, C., Fyrilla, M., Stephanou, A., & Patrikios, I. (2023). DHA/EPA (Omega-3) and LA/GLA (Omega-6) as bioactive molecules in neurodegenerative diseases. *International Journal of Molecular Sciences*. 10.3390/ijms241310717 37445890 10.3390/ijms241310717 PMC10341783

